# Workplace Violence Against Nurses in Psychiatric Hospitals in Oman: A cross-sectional multi-centre study

**DOI:** 10.18295/squmj.7.2023.046

**Published:** 2024-05-27

**Authors:** Maryam Al-Kalbani, Tamadhir Al-Mahrouqi, Siham Al-Shamli, Sathiya Murthi, Naser Al-Balushi, Hamed Al-Sinawi

**Affiliations:** 1 Psychiatry Residency Training Program, Oman Medical Specialty Board, Muscat, Oman; 3 Research Department, Oman Medical Specialty Board, Muscat, Oman; 2 Department of Behavioral Medicine, Sultan Qaboos University Hospital, Sultan Qaboos University, Muscat, Oman

**Keywords:** Workplace, Workplace Violence, Occupational Stress, Working Conditions, Nurses, Psychiatry, Oman

## Abstract

**Objectives:**

This study aimed to assess the prevalence of workplace violence (WPV) against nurses in Oman’s psychiatric hospitals and explore associated factors.

**Methods:**

This cross-sectional study was conducted between October and December 2021 and included all tertiary mental healthcare hospitals in Oman (Al Masarra Hospital and Sultan Qaboos University Hospital, Muscat, Oman). The participants completed a sociodemographic survey and a questionnaire on WPV in the health sector.

**Results:**

A total of 106 participants (response rate = 80.3%) were included in this study. Most were female (52.8%) and Omani (72.6%) and aged 30–39 years. WPV prevalence was high (90.6%), with verbal (86.8%) and physical violence (57.5%) being the most common types. WPV incidents were more frequent on weekdays (26.4%) and during morning shifts (34%), while 81.1% of the nurses worked in shifts and had direct physical contact with patients (83.0%). The majority (92.5%) were aware of standardised WPV reporting procedures and 89.7% confirmed the presence of such procedures in hospitals. WPV was more prevalent among nurses in inpatient wards (*P* = 0.047).

**Conclusion:**

WPV against nurses in Omani psychiatric hospitals is alarmingly high. Future studies should investigate contributing factors among healthcare providers and emphasise violence prevention by providing staff nurses with effective training to handle violent incidents involving psychiatric patients.

**Advances in Knowledge**

- *Workplace violence (WPV) against nurses in tertiary psychiatry hospitals in Oman was high with verbal and physical violence being the most common types.*
- *This study also explores the factors associated with workplace violence affecting mental healthcare nurses.*
- *WPV was predominantly experienced by nurses in inpatient wards. Direct contact with patients and working with fewer staff members correlated with a higher prevalence of violence.*
- *The level of worry about violence was significantly associated with bullying.*

**Applications to Patient Care**

- *The alarming results of this study and its implications call for multilevel actions to reduce workplace violence in psychiatry services across Oman.*
- *This study’s results may contribute to the establishment of safe working environments for staff nurses in psychiatry hospitals, ultimately enhancing the healthcare provided to patients.*

Workplace violence (WPV) has been defined by the European Commission as “incidents where staff are abused, threatened or assaulted in circumstances related to their work, including commuting to and from work, involving an explicit or implicit challenge to their safety, well-being or health”.^1^ According to the World Health Organization (WHO), the definition of violence is “the intentional use of physical force or power, threatened or actual, against oneself, another person or against a group or community, that either result in or has a high likelihood of resulting in injury, death, psychological harm, maldevelopment or deprivation”.^2^

WPV has been a persistent problem, which is underestimated and generally disregarded by the public and professional organisations.^3^ On an international level, it has been noticed that the highest number of these assaults are directed towards healthcare workers.^4^ The Occupational Safety and Health Administration stated that from 2002 to 2013, incidents of serious WPV were 4 times more common in the field of healthcare than in other industries on average.^5^ A systematic review study by Iozzino *et al*. found that nearly 1 in 5 patients admitted to acute psychiatric units may engage in violent behaviour.^6^ In acute psychiatric settings, staff nurses frequently interact closely with patients, placing them at a higher risk of experiencing violent incidents.^7^

Several studies in different parts of the world have explored WPV. A study conducted in Switzerland and published in 2021 showed that 73% of nurses reported facing verbal violence, 63% experienced violence against property, 40% verbal sexual violence, 28% physical violence, 14% physical sexual violence and almost 30% had suffered a serious assault in their lifetime in the workplace.^8^ In Jordan, a study published in 2018 revealed that more than three-quarters of mental health nurses experienced WPV during their tenure in psychiatric hospitals and patients were the main source of violence.^9^ A systematic review from Korea in 2022 showed that the prevalence of WPV ranged from 11.4–97.6%, and nurse-related factors such as age, gender, marital status, education and work shift were associated with the occurrence of WPV.^10^ A similar systematic review conducted in China in 2022 also reached a similar conclusion, identifying potential factors contributing to violence against psychiatric nurses including age, gender, educational background, years of experience and working hours.^11^

Studies on WPV have also been carried out in the Gulf Cooperation Countries. For example, in Saudi Arabia, a study examined the prevalence of WPV among nurses working in mental health hospitals. It showed that the WPV against nurses was approximately 90%, of which 57.7% included both physical and verbal abuse.^3^ Another cross-sectional study published in 2022 in Saudi Arabia in Al-Taif Mental Health Hospital revealed that both nurses and students were generally assaulted by patients or their family members. Nurses mostly handled these situations themselves whereas students often called for help and/or activated alarms.^12^ In Oman, a 2020 study by Al Maskari *et al*. examined the prevalence of violence directed at emergency department nurses. The study revealed that a significant majority of nurses, accounting for 87.4%, experienced some form of violence. Notably, non-physical violence, which constituted 84.5% of the cases, was more prevalent than physical violence, which accounted for 18.4% of the cases.^13^

WPV has direct and detrimental effects, including reduced job satisfaction, increased burnout, feelings of humiliation, guilt, emotional stress, job dissatisfaction and higher staff turnover.^14^ A cross-sectional study involving Chinese physicians examined how WPV influenced job satisfaction, job burnout and turnover intention while also investigating the mediating role of social support. Data were gathered from 9 tertiary hospitals in China, and the results indicated that WPV was positively correlated with turnover intention (r = 0.238; *P* <0.01) and job burnout (r = 0.150; *P* <0.01) while being negatively associated with job satisfaction (r = −0.228; *P* <0.01) and social support (r = −0.077; *P* <0.01). Social support was a partial mediator between WPV and job satisfaction, as well as burnout and turnover intention.^15^

In Pakistan, a study investigated how WPV affected the mental health of emergency physicians. The findings revealed a significant impact, with 1 in 6 physicians screening positive for post-traumatic stress disorder (PTSD) and 2 in 5 experiencing current anxiety and depression. In addition, those who reported to have suffered physical attacks were 6.7 times more likely to exhibit PTSD symptoms. Additionally, the study identified high rates of burnout among physicians, with 42.4% experiencing emotional exhaustion and 72.9% reporting depersonalisation.^16^

The purpose and rationale for studying WPV among nurses in psychiatric hospitals is multifaceted. First and foremost, the safety and well-being of nurses are of paramount importance. Psychiatric hospitals, by nature, house patients with a wide range of mental health conditions, some of which may lead to aggressive or violent behaviour. Therefore, understanding the prevalence and correlates of WPV in this specific setting is crucial for ensuring the safety of the nursing staff.^14^ Furthermore, WPV has many negative consequences as described earlier, such as psychological harm, stress and reduced job satisfaction, which can ultimately affect the quality of patient care.^14^ A systemic review and a meta-analysis conducted by Wang *et al*. found that nurses exposed to WPV had 2.13 and 2.25 times higher odds of experiencing PTSD and burnout compared to those not exposed, even after adjusting for confounding factors.^17^

Research about the prevalence and contributing factors of WPV against nurses within the mental healthcare context has not yet been initiated in Oman. This study aimed to assess the frequency of WPV targeting nurses across all tertiary psychiatric hospitals in Oman while also delving into the factors that are associated with such incidents. Recognising the prevalence and comprehending the patterns of violence within psychiatric departments are crucial steps towards formulating effective policies and strategies for reducing and managing exposure to WPV.^6^

## Methods

This cross-sectional study was conducted between October and December 2021 at two tertiary mental healthcare hospitals in Oman: Al Masarra Hospital (AMH) and Sultan Qaboos University Hospital (SQUH), Muscat, Oman; these hospitals constitute the entirety of the tertiary mental healthcare hospitals in Oman. The psychiatry services in AMH is a tertiary care mental health hospital and includes general adult psychiatry, geriatric psychiatry, child and adolescent psychiatry, drug and substance abuse and forensic psychiatry. The SQUH psychiatry department offers both outpatient and inpatient services, encompassing general adult psychiatry, geriatric psychiatry, child and adolescent psychiatry and consultation-liaison psychiatry.

All registered nurses working for the mental health services at the study sites were invited to participate in this study. This was a self-administered online questionnaire sent to all nursing staff via the institution’s email system. After one week, an electronic reminder was sent. All participants who signed the informed electronic consent form and completed the questionnaire were included in the study. Non-clinical staff nurses, such as those who work in administrative roles, quality assurance and infection control, were excluded from the study.

Sociodemographic data was collected via a questionnaire that asked for information regarding the participant’s age, gender, marital status, status as a citizen or resident of Oman, educational level and years of experience and the departments and institutions of the hospital where they worked.

‘Workplace violence in the health sector’ is a validated self-administered questionnaire developed by the Joint Program on Workplace Violence in the Health Sector of the International Labour Office, the International Council of Nurses, the WHO and Public Services International.^18^ This questionnaire has been used in several studies from different regions, including countries with similar healthcare systems and socioeconomic features to Oman, such as Saudi Arabia.^3^ In Oman, Al-Maskari *et al*. used this questionnaire to examine WPV among emergency department nurses.^13^ The current study modified the questionnaire to meet the study objectives and the cultural context of Oman after obtaining permission from the WHO Department of Strategy, Policy and Information (permission request #369206).

To ensure its originality and avoid language-based distortions in measuring WPV, the questionnaire was presented in English. The questionnaire has 32 items divided into two sections: the first section (physical workplace violence) included questions to evaluate exposure to or witnessing physical violence in the workplace in the past 12 months. These questions were followed by questions on the frequency and characteristics—how the incidents were dealt with and the consequences of the most recent incident. This section of the questionnaire included 11 questions. The second section (psychological workplace violence) included questions to evaluate verbal abuse, bullying/mobbing and racial harassment in the workplace in the past 12 months. These questions were followed by questions on the frequency and characteristics—how the incidents were dealt with and the consequences of the most recent incident. This section of the questionnaire included 20 items.

In a cover letter attached to the questionnaire, all participants were provided with the definitions of each type of WPV (physical, verbal, bullying and racial harassment), as well as examples of each type of WPV [Supplementary Table 1].

AMH and SQUH had a total of 132 registered nurses working for mental health services during the study period. The sample size was calculated using the OpenEpi programme (Emory University, Atlanta, Georgia, USA). With a type-1 error of 5.0% (alpha = 0.05) and a 95% level of significance, the sample size was determined to achieve a power level of 80.0%, with a design effect of 1. In the existing literature, the prevalence of WPV among mental health services nursing staff is approximately 90%.^3^ As a result, the needed minimum sample size was 68.

Data were analysed using Statistical Package for the Social Sciences (SPSS), Versions 25 and 28 (IBM Corp, Armonk, New York, USA). Continuous variables were summarised using mean ± standard deviation and categorical variables with numbers and percentages. Categorical variables were compared using the Chi-squared test and the *P* value of <0.05 was considered statistically significant.

The procedures followed the ethical guidelines of the Declaration of Helsinki. Electronic written informed consent was obtained from the participants prior to the administration of the questionnaire.

## Results

A total of 106 participants completed questionnaires (response rate = 80.3%). Most of the participants were Omani (72.6%), half of them were females and almost two-thirds were married. The largest proportion had a bachelor’s degree (54.7%). Most of the nurses were working in AMH (74.5%) and in inpatient wards (80.2%); 36.8% had work experience of more than 10 years. The majority were working in shifts (81.1%) with direct physical contact with the patients (83.0%). Approximately 40.6% of the staff were working with patients of both genders and 46.2% were working with male patients. Most of the participants were working with patients from all age groups (53.8%) [Table 1].

On a scale of 1 to 5 (with 5 being ‘very worried’), more than half of the nurses were moderately worried about violence in their workplace (58.4%). Most of the respondents indicated that they were aware of the existence of WPV reporting protocols (92.5%) and were knowledgeable about how to use the standardised WPV reporting procedures in their hospital (89.7%). Those who were encouraged to report WPV (88.2%) indicated hospital management (81.1%) followed by colleagues (17.8%) and family and friends (1.1%) as the sources of encouragement [Table 2].

The prevalence of WPV among nurses was 90.6%. The highest type of violence experienced in the last 12 months was verbal violence (86.8%) followed by physical violence (57.5%).

The majority of physical violence incidents (26.4%) took place during weekdays, with the highest incidences happening during morning shifts (n = 36, 34%) followed by afternoon shifts (n = 27, 25.5%). Patients were the most reported source of all types of violence [Table 3].

There were different ways of responding to different types of WPV, with the majority of staff (n = 34, 32.1%) submitting an incident/accident form, followed by 31.1% telling the person committing the violence to stop and reporting it to a senior staff member. Most staff members exposed to verbal violence (n = 45, 45.5%) told the person to stop but 17.9% took no action against the violence. While the majority of staff who were exposed to physical violence and mobbing thought that the violence could have been prevented, the staff who were exposed to verbal violence and racial harassment did not think that the incident could have been prevented. Although hospital management was identified as the primary source of motivation for reporting WPV, the findings revealed that no steps were taken to probe the underlying reasons for a significant portion of non-physical violence incidents (62.8%, 71.7% and 67.6% for verbal, mobbing and racial harassment, respectively). Approximately 17.9% of those exposed to physical violence, 36.8% to verbal violence and 14.2% to mobbing stated the reason for not reporting the incident as ‘not important’. However, among those exposed to racial harassment, 12.3% felt that reporting was useless. Omani nurses (n = 13, 12.3%) suffered from racial harassment more than non-Omani nurses (n = 7, 6.6%) [Table 4].

WPV was mostly faced by nurses working in inpatient wards (*P* = 0.047). Nurses are more at-risk to face physical violence and bullying. The level of worry about violence was significantly associated with WPV, especially verbal violence and bullying (*P* = 0.072 and 0.023, respectively). In addition, being in direct contact with patients and working with fewer staff members was statistically significant with a higher prevalence of violence (*P* = 0.008 and 0.004, respectively).

## Discussion

Studying WPV among psychiatric nurses is crucial because it has significant repercussions on their mental health and work-related outcomes. Psychiatric nurses exposed to WPV frequently suffer from poor mental health such as anxiety and depressive symptoms and show negative work-related consequences such as an increased intention to leave their jobs.^10^ A study conducted in Chinese psychiatric hospitals among psychiatric nurses revealed that those who experienced WPV had lower quality of life in both their physical and mental well-being domains compared to their counterparts who were not exposed to WPV.^19^ This finding highlights the importance of addressing WPV to safeguard the mental and physical health of psychiatric nurses and ultimately enhance the quality of patient care.

The current study revealed a significant prevalence of WPV against nurses, amounting to 90.6%. This aligns with the results of a systematic review, which included 16 articles published between 2011 and 2020 and identified WPV prevalence ranging from 11.4% to 97.6%.^10^ Moreover, the prevalence of WPV in this study is in line with prior research conducted by Basfr *et al*. and a study by Liu *et al*.^3^^,^^20^[Fig f1-squmj2405-194-202]

Over the 12-month study period, the most frequently reported form of violence encountered by nurses was verbal abuse, affecting 86.8% of participants, followed by physical violence, reported by 57.5%. These results closely align with findings from another study from China involving psychiatric nurses, where a significant majority experienced verbal harassment (78.6%), followed by physical harassment (61.5%) and sexual assault (18.6%).^19^ Another study on healthcare workers in Nepal revealed that patients and their family members were primarily responsible for incidences of physical and verbal violence, while incidents of bullying and mobbing were predominantly instigated by management and fellow staff members.^21^ Similar to previous findings, the current study showed that patients were the most frequent source of violence, with 63.2% reporting physical violence with only one case involving a relative of a patient physically abusing a staff member. This aligns with a study conducted in Saudi Arabia, where violent behaviour was primarily exhibited by the patients themselves (81.3%).^3^

The high prevalence of violence against psychiatric nurses can be attributed to various contributing factors. First, the patients admitted to tertiary care psychiatric hospitals in Oman often present with severe psychiatric symptoms. This could potentially lead to a higher occurrence of violent incidents within inpatient departments. In comparison, incidents of violence in outpatient departments tend to be less frequent due to the typically less severe nature of patients’ illnesses in these settings.^22^

Nurses working in shifts were more likely to experience violence than those who work in outpatient departments with fixed morning schedules. This finding is consistent with that of Niu *et al*., who revealed that Chinese nurses who worked in rotating shifts had an increased risk of physical violence and psychological violence compared to those who worked in regular morning schedules.^7^ In addition, another survey study involving 387 frontline psychiatric nurses in China revealed that working on rotating duty was highly associated with increased WPV.^19^ These combined factors significantly impact the safety and well-being of nurses, necessitating urgent attention and intervention to create safer working conditions and improve patient care.

The current study revealed that nurses with more years of experience have a higher percentage of exposure to violence. Senior nurses are more likely to be assigned to patients with higher disease severity and those that are deemed difficult to handle, which may result in greater exposure to violence than other nurses. This result was also consistent with the finding of Niu *et al.*, who showed that nurses with work experience of 5 to 10 years had an increased risk of psychological violence.^7^ Another study revealed that nurses with 6 to 10 years of work experience had a higher risk of WPV.^23^

The majority of the participants acknowledged the existence of standardised WPV reporting procedures in their hospitals and were aware of how to use them. Those who expressed encouragement to report WPV attributed it to hospital management, a finding in line with a study conducted by Niu *et al*.^7^ Unfortunately, WPV is often ignored as an unfortunate aspect of the job rather than something to be reported.^14^

The current study is the first comprehensive investigation into the prevalence of WPV against psychiatric nurses in Oman. Through multivariate analyses, this study examined the associated factors and correlates of such violence, contributing valuable insights to the existing literature. This study was conducted in tertiary care hospitals covered both inpatient and outpatient settings, allowing for a more thorough understanding of the prevalence and patterns of WPV in psychiatric healthcare. These findings will enable healthcare institutions to identify critical areas of concern and implement targeted interventions to enhance staff safety and patient care in both inpatient and outpatient environments. As a result, this study holds significant implications for fostering a safer and more supportive work environment for psychiatric nurses in Oman.

This study recommends the implementation of strategies to reduce or prevent violence and employ effective reporting systems for WPV to reduce the risk of violence.^24^ Hospitals should develop an effective system to report WPV so that the incidence and root causes can be understood, and therefore strategies can be implemented to reduce violence towards staff. Management should handle all reported cases promptly as a sentinel event and conduct a risk assessment accordingly, with a subsequent action plan to reduce further occurrences.

A reporting system will provide important information for the effectiveness of the improvement efforts and can inform organisational policy. Secondly, the curriculum for psychiatric nurse training in Oman should ensure adequate skills training for screening patients with potential for violence and implementing strategies to reduce the risk for violence including handling patients’ emotional and behavioural problems such as de-escalation techniques. Wu *et al*. showed that training based only on lectures is less effective in preventing WPV compared to WPV training programmes in hospital settings based on interactive and dynamic learning methods for the workers.^25^ For example, teaching strategies such as small-group learning, interactive learning and simulation exercises may be applied during training in medical schools. Finally, additional studies investigating the long-term psychological and physical negative effects of WPV on healthcare workers would be of great value, as this could add to the knowledge about the associations between WPV and adverse effects. Some examples are PTSD, depression, anxiety, burn-out and turnover. Supportive counselling services should be provided for psychiatric nurses who encounter WPV.

This study was subject to several limitations. First, the cross-sectional design of the survey restricts the study’s ability to establish causal relationships between violence and its correlates. Longitudinal studies would provide more robust insights into the dynamics of WPV among nurses. Lastly, relying solely on self-reported measures of violence might result in a reluctance to share private information or misunderstandings regarding the definition of violence. To address this limitation, future studies could consider incorporating qualitative assessments such as semi-structured interviews to gain a more comprehensive understanding of WPV.

## Conclusion

The current study found that WPV against nurses in Omani psychiatric hospitals is high. There is a pressing concern for the well-being of these healthcare professionals. Addressing this critical issue necessitates prioritised investments in preventing WPV. Regular and comprehensive training programmes should be implemented for all healthcare workers in mental hospitals throughout Oman. These training initiatives should equip staff with practical strategies to handle and respond to violent incidents involving psychiatric patients, promoting a safer and more supportive working environment. As a result, the quality of healthcare services provided to patients can significantly improve, ensuring the well-being of both medical professionals and those seeking care.

## Supplementary Information



## Figures and Tables

**Figure 1 f1-squmj2405-194-202:**
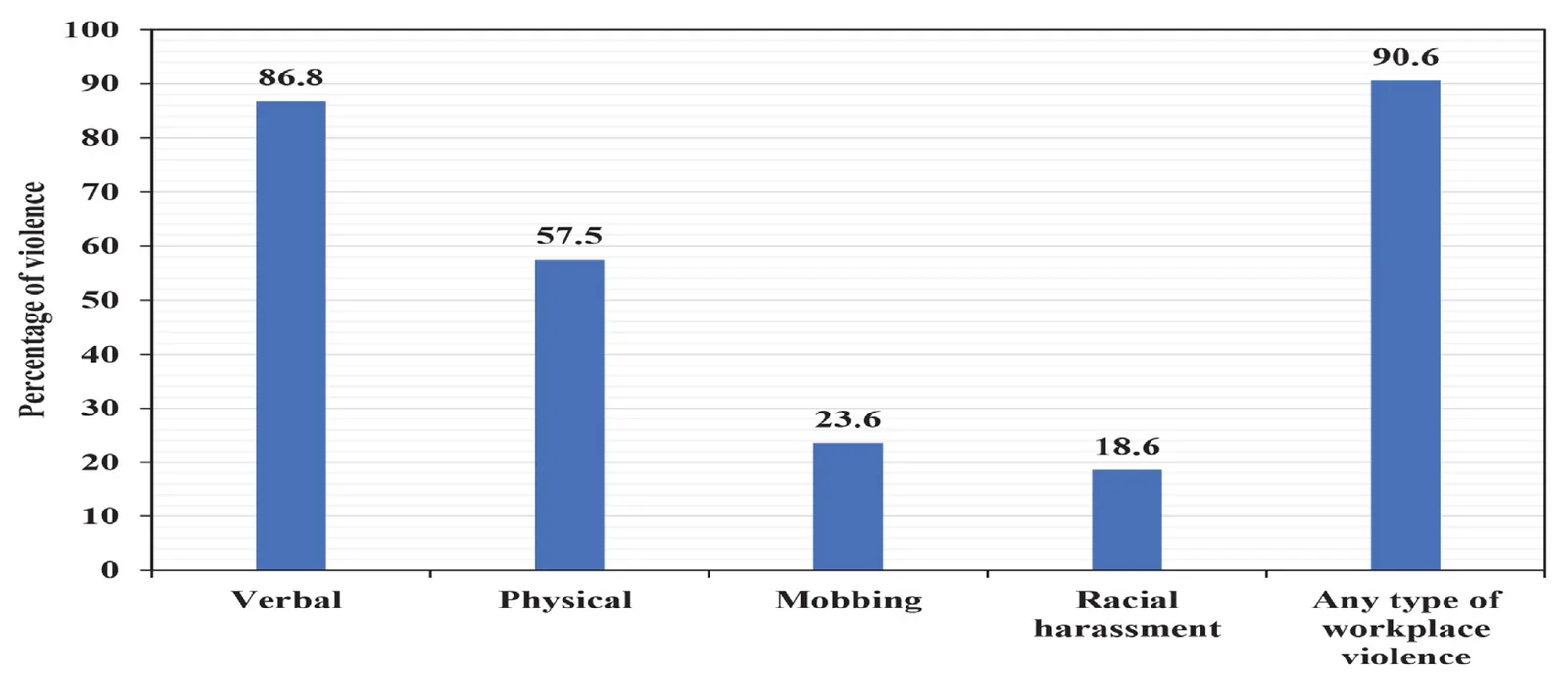
Prevalence of each type of workplace violence.

**Table 1:** Characteristics of nurses exposed to physical violence and verbal abuse at psychiatric hospitals in Oman (N = 106)

Characteristic	n (%)		*P* value	n (%)		*P* value
	Physical violence			Verbal abuse		
	Yes (n = 61)	No (n = 45)		Yes (n = 92)	No (n = 14)	
**Gender**			χ^2^ (1, n = 106) = 0.09, *P* = 0.845			χ^2^ (1, n = 106) = 0.85, *P* = 0.402
Male	28 (45.9)	22 (48.9)		45 (48.9)	5 (35.7)	
Female	33 (54.1)	23 (51.1)		47 (51.1)	9 (64.3)	
**Marital status**			χ^2^ (1, n = 106) = 0.56, *P* = 0.496			χ^2^ (1, n = 106) = 2.42, *P* = 0.180
Single	16 (26.2)	9 (20.0)		24 (26.1)	1 (7.1)	
Married	45 (73.8)	36 (80.0)		68 (73.9)	13 (92.9)	
**Nationality**			χ^2^ (1, n = 106) = 0.02, *P* = 1.000			χ^2^ (1, n = 106) = 0.57, *P* = 0.523
Omani	44 (72.1)	33 (73.3)		68 (73.9)	9 (64.3)	
Non-Omani	17 (27.9)	12 (267)		24 (26.1)	5 (35.7)	
**Education**			χ^2^ (2, n = 106) = 1.19, *P* = 0.551			χ^2^ (2, n = 106) = 2.61, *P* = 0.271
Diploma in nursing	27 (44.3)	17 (37.8)		40 (43.5)	4 (28.6)	
Bachelor’s	31 (50.8)	27 (60.0)		48 (52.2)	10 (71.4)	
Master’s	3 (4.9)	1 (2.2)		4 (4.3)	-	
**Institution**			χ^2^ (1, n = 106) = 0.04, *P* = 1.000			χ^2^ (1, n = 106) = 0.08, *P* = 0.750
SQUH	16 (26.2)	11 (24.4)		23 (25.0)	4 (28.6)	
AMH	45 (73.8)	34 (75.6)		69 (75.0)	10 (71.4)	
**Working department**			χ^2^ (3, n = 106) = 7.97, P = 0.047			χ^2^ (3, n = 106) = 2.85, P = 0.416
Inpatient ward	48 (78.7)	37 (82.2)		74 (80.4)	11 (78.6)	
Outpatient	6 (9.8)	2 (4.4)		6 (6.5)	2 (14.3)	
ED	6 (9.8)	1 (2.2)		7 (7.6)	-	
Rehabilitation ward	1 (1.6)	5 (11.1)		5 (5.4)	1 (7.1)	
**Work experience in years**			χ^2^ (3, n = 106) = 0.48, *P* = 0.923			χ^2^ (3, n = 106) = 4.42, *P* = 0.220
<1	2 (3.3)	2 (4.4)		2 (2.2)	2 (14.3)	
1–4	18 (29.5)	15 (33.3)		28 (30.4)	5 (35.7)	
5–10	17 (27.9)	13 (28.9)		26 (28.3)	4 (28.6)	
>10	24 (39.3)	15 (33.3)		36 (39.1)	3 (21.4)	
**Routine direct physical contact**			χ^2^ (1, n = 106) = 7.87, *P* = 0.008			χ^2^ (1, n = 106) = 1.54, *P* = 0.251
Yes	56 (91.8)	32 (71.1)		78 (84.8)	10 (71.4)	
No	5 (8.2)	13 (28.9)		14 (15.2)	4 (28.6)	
**Patients**’ gender*			χ^2^ (2, n = 106) = 0.316, *P* = 0.854			χ^2^ (3, n = 106) = 1.93, *P* = 0.381
Male	28 (45.9)	21 (46.7)		44 (47.8)	5 (35.7)	
Female	9 (14.8)	5 (11.1)		13 (141)	1 (7.1)	
Both	24 (39.3)	19 (42.2)		35 (38.0)	8 (57.1)	
**Do you work in shifts?**			χ^2^ (1, n = 106) = 1.57, *P* = 0.315			χ^2^ (1, n = 106) = 0.22, *P* = 1.000
Yes	47 (77.0)	39 (86.7)		74 (80.4)	12 (85.7)	
No	14 (23.0)	6 (13.3)		18 (19.6)	2 (14.3)	
**Number of staff members in the same work setting**			2 (2, n = 106) = 10.84, *P* = 0.004			χ^2^ (2, n = 106) = 1.55, *P* = 0.462
1–4	25 (41.0)	25 (55.6)		44 (47.8)	6 (42.9)	
4–8	35 (57.4)	14 (31.1)		43 (46.7)	6 (42.9)	
>8	1 (1.6)	6 (13.3)		5 (5.4)	2 (14.3)	

SQUH = Sultan Qaboos University Hospital; AMH = Al Masarra Hospital; ED = emergency department.

* Nurses who experienced violence from male patients only, female patients only, or both genders.

**Table 2:** Questions regarding the utilisation of the reporting system in psychiatry hospitals in Oman (N = 106)

Question	n (%)
**How worried are you? (n = 106)**	
1	9 (8.5)
2	23 (21.7)
3	38 (35.8)
4	24 (22.8)
5	12 (11.3)
**Any procedures to report? (n = 106)**	
Yes	98 (92.5)
No	8 (7.5)
**Do you know how to use them? (n = 97)**	
Yes	87 (89.7)
No	10 (10.3)
**Any encouragement to use the reporting system? (n = 102)**	
Yes	90 (88.2)
No	12 (11.8)
**Who encouraged you to report? (n = 90)**	
Management/employer	73 (81.1)
Colleagues	16 (17.8)
Family members/friends	1 (1.1)

**Table 3:** Prevalence of each type of workplace violence (N = 106)

Type of workplace violence	n (%)	95% CI
Physical	61 (57.5)	47.57–67.09
Verbal	92 (86.8)	78.83–92.59
Mobbing	25 (23.6)	15.88–32.82
Racial harassment	20 (18.9)	11.92–27.62
General	96 (90.6)	83.3–95.38

CI = confidence interval

**Table 4:** Participants’ response to workplace violence at psychiatric hospitals in Oman (N = 106)

Response to different types of workplace violence	n (%)			
	Physical	Verbal	Bullying/mobbing	Racial harassment
**How did you respond to the incident?**				
Took no action	7 (6.6)	28 (26.4)	19 (17.9)	11 (10.4)
Tried to pretend it never happened	10 (9.4)	20 (18.9)	8 (7.5)	5 (4.7)
Told the person to stop	33 (31.1)	45 (42.5)	13 (12.3)	6 (5.7)
Tried to defend myself physically	28 (26.4)	11 (10.4)	1 (0.9)	2 (1.9)
Told friends/family	8 (7.5)	3 (2.8)	3 (2.8)	2 (1.9)
Sought counselling	4 (3.8)	4 (3.8)	1 (0.9)	-
Told a colleague	24 (22.6)	23 (21.7)	12 (11.3)	6 (5.7)
Reported it to a senior staff member	33 (31.1)	33 (31.1)	15 (14.2)	13 (12.3)
Transferred to another position	6 (5.7)	3 (2.8)	-	1 (0.9)
Completed incident/accident form	34 (32.1)	17 (16.0)	-	3 (2.8)
Pursued prosecution	4 (3.8)	1 (0.9)	6 (5.7)	1 (0.9)
Completed a compensation claim	1 (0.9)	2 (1.9)	1 (0.9)	2 (1.9)
Other	6 (5.7)	3 (2.8)	-	1 (0.9)
**Do you think the incident could have been prevented?**				
Yes	37 (56.9)	36 (38.3)	26 (59.1)	16 (45.7)
No	28 (43.1)	58 (61.7)	18 (40.9)	19 (54.3)
**Was any action taken to investigate the causes of the mobbing/bullying?**				
Yes		35 (37.2)	13 (28.3)	11 (32.4)
No		59 (62.8)	33 (71.7)	23 (67.6)
**What were the reasons for not reporting (if any)?**				
It was not important	19 (17.9)	39 (36.8)	15 (14.2)	11 (10.4)
Felt ashamed	2 (1.9)	5 (4.7)	4 (3.8)	2 (1.9)
Felt guilty	3 (2.8)	2 (1.9)	5 (4.7)	2 (1.9)
Afraid of negative consequences	10 (9.4)	8 (7.5)	6 (5.7)	5 (4.7)
Useless	7 (6.6)	30 (28.3)	13 (12.3)	13 (12.3)
Did not know who to report to	2 (1.9)	3 (2.8)	1 (0.9)	-
Other	5 (4.7)	10 (9.4)	2 (1.9)	2 (1.9)
